# Is Vestibular Self-Motion Perception Controlled by the Velocity Storage? Insights from Patients with Chronic Degeneration of the Vestibulo-Cerebellum

**DOI:** 10.1371/journal.pone.0036763

**Published:** 2012-06-15

**Authors:** Giovanni Bertolini, Stefano Ramat, Christopher J. Bockisch, Sarah Marti, Dominik Straumann, Antonella Palla

**Affiliations:** 1 Department of Neurology, Zurich University Hospital, Zurich, Switzerland; 2 Department of Computer Science, University of Pavia, Pavia, Italy; 3 Department of Otorhinolaryngology, Head and Neck Surgery, Zurich University Hospital, Zurich, Switzerland; 4 Department of Ophthalmology, Zurich University Hospital, Zurich, Switzerland; University of Regensburg, Germany

## Abstract

**Background:**

The rotational vestibulo-ocular reflex (rVOR) generates compensatory eye movements in response to rotational head accelerations. The velocity-storage mechanism (VSM), which is controlled by the vestibulo-cerebellar nodulus and uvula, determines the rVOR time constant. In healthy subjects, it has been suggested that self-motion perception in response to earth-vertical axis rotations depends on the VSM in a similar way as reflexive eye movements. We aimed at further investigating this hypothesis and speculated that if the rVOR and rotational self-motion perception share a common VSM, alteration in the latter, such as those occurring after a loss of the regulatory control by vestibulo-cerebellar structures, would result in similar reflexive and perceptual response changes. We therefore set out to explore both responses in patients with vestibulo-cerebellar degeneration.

**Methodology/Principal Findings:**

Reflexive eye movements and perceived rotational velocity were simultaneously recorded in 14 patients with chronic vestibulo-cerebellar degeneration (28–81yrs) and 12 age-matched healthy subjects (30–72yrs) after the sudden deceleration (90°/s2) from constant-velocity (90°/s) rotations about the earth-vertical yaw and pitch axes. rVOR and perceived rotational velocity data were analyzed using a two-exponential model with a direct pathway, representing semicircular canal activity, and an indirect pathway, implementing the VSM. We found that VSM time constants of rVOR and perceived rotational velocity co-varied in cerebellar patients and in healthy controls (Pearson correlation coefficient for yaw 0.95; for pitch 0.93, p<0.01). When constraining model parameters to use the same VSM time constant for rVOR and perceived rotational velocity, moreover, no significant deterioration of the quality of fit was found for both populations (variance-accounted-for >0.8).

**Conclusions/Significance:**

Our results confirm that self-motion perception in response to rotational velocity-steps may be controlled by the same velocity storage network that controls reflexive eye movements and that no additional, e.g. cortical, mechanisms are required to explain perceptual dynamics.

## Introduction

In the absence of visual cues, it is mainly the vestibular system providing the brain with a sense of body and spatial orientation. The semicircular canals detect rotational movements and the otolith organs detect translational movements as well as the orientation of the head relative to gravity. Specifically, the semicircular canals sense angular and the otolith organs sense linear acceleration. Thus, when the head moves at a constant angular velocity, after an initial deflection, the cupula of the sensory organ passively returns to its resting position resulting in a decay of the firing rate of the vestibular nerve and, therefore, in a decrease of movement sensation. The semicircular canal cupular time constant has been estimated to range between 3 and 7 seconds [1], [2], [3], [4], [5], [6], [7], [8], [9]. The vestibular nystagmus that emerges from a constant-velocity rotation in darkness (i.e. the compensatory eye movements evoked by a head rotation), however, outlasts the duration of the semicircular canal input. It is currently believed that a central brainstem and cerebellar network, known as the velocity storage mechanism, prolongs the semicircular canal afferent signal, extending the duration of reflexive eye movements and improving the compensatory response to low-frequency head rotations [8], [10], [11].

Although the concept of the velocity storage mechanism has been developed based on reflexive eye movements, Okada et al. have suggested that this velocity storage mechanism is also present at higher-level cortical processes such as in rotational self-motion perception [12]. Our recent observations in healthy human subjects support such hypothesis as we found that self-motion perception after the sudden deceleration from constant-velocity rotations (i.e. angular velocity steps) could be modeled using the same central velocity storage element as the rotational vestibulo-ocular reflex (rVOR) [13]. In contrast to the current opinion [12], [14], therefore, our results suggested that no additional, e.g. cortical, mechanisms are required to explain the perceptual dynamics in healthy subjects and, thus, corroborate the hypothesis that the velocity storage mechanism plays an important role also in self-motion perception. In the present study, we aimed at further investigating this hypothesis. Specifically, we speculated that if our hypothesis about a common velocity storage mechanism in the rVOR and in rotational self-motion perception is correct, a dysfunction of velocity storage would result in similar changes in reflexive and perceptual responses.

**Table 1:** Diagnosis, most prominent clinical and MRI findings in cerebellar patients.

Patient No., gender, age (y)	Diagnosis	Main clinical findings	Brain MR
1, m, 66	SAOA	DBN, GEN, SP, GA	slight atrophy of VestCb
2, m, 70	SAOA	DBN, GEN, SP, GA	moderate atrophy of V
3, m, 81	SAOA	DBN, GEN, saccadic SP, GA	severe atrophy of VestCb
4, f, 34	most probably sporadic	GA, LA, upper limb rebound phenomenon	slight atrophy of V and cerebellar hemispheres
5, m, 78	SAOA	DBN, GEN, GA, (SP), (LA)	severe atrophy of VestCb and cerebellar hemispheres
6, f, 57	probably immune-mediated (Glutenataxia)	DBN, GA, SP	no atrophy
7, m, 66	SAOA	(DBN), SP, GEN, Dysarthria, (GA), (LA)	severe atrophy of V and cerebellar hemispheres
8, m, 35	hereditary or sporadic	GA, (LA), Dysarthria, (SP)	slight atrophy of V and cerebellar hemispheres
9, m, 38	ADCA III	DBN, GEN, GA, Dysarthria	atrophy of V and cerebellar hemispheres
10, m, 45	ADCA III	(DBN), SP, GEN, GA, LA, Dysarthria	severe atrophy of V and cerebellar hemispheres
11, m, 28	most probably hereditary	GA, (LA), (SP)	severe atrophy of V and cerebellar hemispheres
12, m, 39	probably ADCA III	DBN, GEN, SP	slight atrophy of V and cerebellar hemispheres
13, f, 66 *	SAOA	(DBN), GEN, SP, ocular flutter, (GA)	slight atrophy of VestCb, cerebellar hemispheres, and colliculus superior
14, m, 51 *	most probably ADCA III	(DBN), GEN, saccadic SPEM, GA, (LA)	slight atrophy of V and cerebellar hemispheres

Definitive or suspected diagnosis, most prominent clinical and MRI findings in the 14 patients studied. Sporadic adult onset ataxia (SAOA); Autosomal-dominantly inherited cerebellar ataxia type III (ADCA III); Downbeat nystagmus (DBN); Gait ataxia (GA); Horizontal gaze evoked nystagmus (GEN); Limb ataxia (LA); Impaired horizontal smooth pursuit eye movements (SP); Vermis (V); Vestibulo-cerebellum (VestCb). Patient No 9 and No 10 are brothers. *: two patients only tested during earth-vertical yaw axis, because of reported motion sickness during earth-vertical pitch rotations.

The velocity storage mechanism is modulated by the vestibulo-cerebellum, specifically the nodulus and ventral uvula [15]. Lesions of these structures impair the ability to realign the eye velocity vector towards the gravito-inertial acceleration vector, i.e. the ability to transform sensory signals encoded in a head-fixed reference frame into a gravitational (i.e. spatially linked) reference frame, a behavior typically attributed to the velocity storage mechanism [16], [17], [18], [19], [20], [21], [22], [23], [24], [25], [26], [27], [28]. The influence of the nodular and uvular structures on the velocity storage activity regarding the control of the time constant of rVOR responses, however, is less clear. Large nodular lesions in monkey, for example, lengthen the overall duration of horizontal rVOR responses, while more circumscribed medial lesions, apparently, slightly shorten them [15], [17], [23], [29]. Similarly, ambiguous findings are reported in humans where even in comparable nodular lesions due to ischemic strokes, some authors observed prolonged horizontal rVOR responses during earth-vertical axis rotations [26], while others found the rVOR duration ranging within normal limits [27], [28]. It has been suggested that these discrepancies resulted from a heterogeneous distribution pattern of affected vestibulo-cerebellar structures [23].

To investigate our hypothesis, namely whether the rVOR and rotational self-motion perception share a common velocity storage mechanism, we decided to explore perceptual and reflexive eye movement responses in patients with midline cerebellar lesions. Even though we could not predict the effect of the cerebellar lesions on the duration of both responses, i.e. whether we would find a prolongation or shortening of the responses, we, nevertheless, expected to find a correlation of both responses in the case of a common velocity storage mechanism. Alternatively, the lack of correlation would imply that vestibular signals for rotational self-motion perception are further processed, possibly at a more rostral level in the central nervous system.

## Methods

### Subjects

14 patients (4 females; mean age 53 yrs, range 28–81yrs) with chronic degeneration of the vestibulo-cerebellum due to hereditary or sporadic disease (see Table 1) and 12 age-matched healthy subjects (5 females; mean age 56 yrs, range 30–72 yrs) participated in the study. Informed consent of all participants was obtained in written form after full explanation of the experimental procedure. The protocol was approved by the Ethics Committee of the Canton of Zurich, Switzerland (Protocol N°E−33/2007), and was in accordance with the ethical standards laid down in the 1964 Declaration of Helsinki for research involving human subjects.

### Experimental Setup

Participants were seated on a turntable with three servo-controlled motor-driven axes (prototype built by Acutronic, Switzerland). They were positioned so that the intersection of the inter-aural and naso-occipital axes was at the intersection of the three axes of the turntable. The head was restrained with an individually molded thermoplastic mask (Sinmed BV, Reeuwijk, The Netherlands). Pillows and safety belts minimized movements of the body.

#### Recording of rotational self-motion perception

Participants were asked to turn a lever attached to a potentiometer that was fixed to the chair. The instruction was to match the rate of lever spinning with the perceived rotational velocity and to stop spinning when the rotation was not felt anymore (for details about instructions see [13]). To avoid additional sensory cues possibly biasing ‘pure’ vestibular rotatory sensation (e.g. auditory cues and/or proprioceptive cues due to chair vibration and/or airflow, which might prolong the vestibular induced spinning sensation during constant velocity rotation) white noise was delivered through headphones and only postrotatory responses (i.e. data collected after stopping the turntable) were analyzed [12]. Experimental instructions were always given by the same experimenter (A.P.) to guarantee consistent information among all participants.

#### Recording of eye movements

Three-dimensional eye movements were recorded monocularly with scleral search coils (Skalar Instruments, Delft, Netherlands) after anesthetizing the conjunctiva with 0.4% Oxybuprocaine. Search coil annuli were calibrated with a method described elsewhere [30]. A turntable-fixed aluminum coil frame (side length 0.5 m) surrounded the head and generated three orthogonal digitally synchronized magnetic wave fields of 80, 96, and 120 kHz. Technical details about data acquisition were described previously [13].

**Figure 1:**
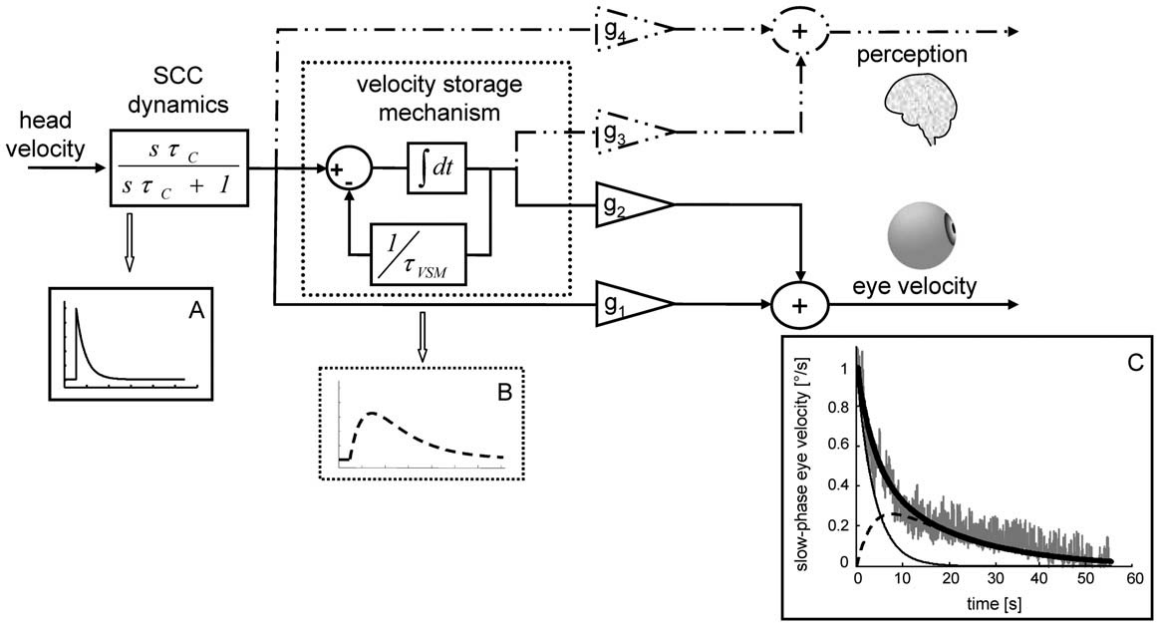
Proposed model structure according to the Raphan, Cohen, and Matsuo model with corresponding modifications for self-motion perception. Block diagram representation of the velocity storage model as previously developed for the rotational vestibulo-ocular reflex (solid lines) [10], [33] and recently modified [13] for rotational self-motion perception (dashed-dotted lines) under the assumption of a common central processing (see text for details). Insert A: Example of the output of the block accounting for the semicircular canal (SCC) dynamics. Insert B: Example of the output of the block representing the central velocity storage mechanism. Insert C: Output of the model (bold black line) generating a curve that best fits slow-phase eye velocity responses (gray traces) of the rotational vestibulo-ocular reflex. The thin and dashed black lines in insert C represent the two components (SCC and velocity storage mechanism) generating the overall data fit (bold line). g_1−4_: gains, i.e. strength or weight of individual pathway contributions.

### Experimental Procedure

Participants were rotated in complete darkness about the earth-vertical axis while seated upright (yaw rotation with predominant horizontal semicircular canal activation) or lying on their left side (pitch rotation with predominant vertical semicircular canal activation). Two patients (indexed as 13 and 14 in Table 1) were rotated about the earth-vertical yaw axis only due to motion sickness during earth-vertical pitch rotations. Data of single participants was obtained during a single session recorded at a determined day.

Within the same session, recordings were restricted to four trials of yaw and four trials of pitch rotations, as habituation of the rotational vestibulo-ocular reflex and of the sensation of self-rotation takes place after ten consecutive trials of steps of angular velocity about the yaw axis [31]. By restricting the number of trials, we also avoided effects of decreasing alertness. Because of this trial restriction and in order to investigate the reproducibility of the perceptual response, however, passive rotations (angular velocity: 90°/s; acceleration and deceleration: 90°/s^2^; duration: 90 s) were delivered only in counterclockwise direction (i.e. positive rotation about the upward pointing space-fixed z-axis according to the right-hand rule [32]). This corresponded to a participant’s backward rotation when lying on the left side. Postrotatory recording in darkness continued until nystagmus (leftward beating in upright position and upward beating in 90° left ear down position) decayed to zero and participants indicated that their perception of rotational motion had vanished. Yaw and pitch rotation trials were pseudo-randomly intermingled for each participant. Between rotation trials, a 30 s to 1 minute break was given with the test room illuminated.

### Data analysis

#### Parameter estimation

A model previously developed for monkeys representing the step responses of the rotational vestibulo-ocular reflex (rVOR) was fitted to our data [10], [33]. Figure 1 (solid lines) provides a graphical representation of this model together with an example of the slow-phase eye velocity responses of one cerebellar patient (indexed as 8 in Table 1) to one trial of earth-vertical yaw rotation (gray trace in insert C). The function that generates the curve that best fits slow-phase eye velocity (bold trace in insert C) can be divided into two components. The first, accounting for the semicircular canal activity, is a single exponential with a time constant *τ_C_* and is shown in the figure by solid thin traces (see inserts A and C). The second component of the function represents the central processing called the velocity storage mechanism and is the difference of two exponentials with two different time constants, *τ_C_* and *τ_VSM_*. It is depicted as dashed traces (see inserts B and C).

The mathematical function describing the curve fit, i.e. the step response of the rVOR is:
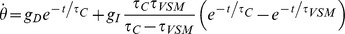
where *τ_C_* and *τ_VSM_* are the semicircular canal and velocity storage time constants, respectively, and *g_D_* and *g_I_* are the gains, i.e. the strength or weight of the peripheral and central contributions (for further details on the mathematical description of the model see [13], [34]). All parameter were iteratively optimized using a nonlinear least-squares algorithm. In order to minimize the impact of approximating a ‘pure’ step response by the actual 90°/s^2^ ramp response used in this study, the end of chair deceleration phase was set at *t = 0* and used as first data point of the data fitting procedure.

In a previous study in healthy subjects we demonstrated that the same function could be used to fit perceived rotational velocity during step responses [13]. Notably, the accuracy of the fit did not decrease when restraining the model to use the same τ_C_ and τ_VSM_ for both slow-phase eye velocity and perceived rotational velocity, suggesting the existence of a common – or at least an equivalent – central processing of reflexive eye movements and self-motion perception. The dashed-dotted lines in figure 1 represent the assumption that self-motion perception and eye movements share a common central processing. In the following, the model will be applied to our data without and with the constraint of a common central processing, i.e. by letting *τ_VSM_* free to change or by using the same *τ_VSM_*. Note that the assumption about the model constraint holds only for the time constants *τ_C_* and *τ_VSM_*, whereas *g_D_* and *g_I_*, i.e. the relative weighting of the peripheral and central pathways, will be left free to change. This is justified since the two gains do not model the time constant substitution accomplished by the velocity storage mechanism, but just the balancing between the velocity storage contribution and the direct semicircular canals signal. Even assuming a common velocity storage, this weighting can easily be different for reflexes and perception as it can occur independently from the velocity storage processing.

#### Statistical analysis

Normal distribution of time constants and gains of each participant was evaluated using Lilliefors test. Pearson correlation coefficients were used for co-variance analysis of *τ_VSM_* estimates resulting from the model fit of slow-phase eye velocity and perceived rotational velocity without the ‘common central processing’ parameter constraint; p values less than 0.05 were considered significant. A measure of the goodness of fit of the model with and without the *τ_VSM_* constraint was, furthermore, provided by the variance-accounted-for technique [35]. Finally, Bayesian information criterion was computed as a measure of the adequacy of model complexity [36]. Whereas the variance-accounted-for measure provides a goodness of fit criterion that is independent of the number of parameters in the fitting model, the Bayesian information criterion takes the number of parameters into account, ‘penalizing’ more complex models for the over-fitting. Variance-accounted-for values should be larger to indicate a superior fit, whereas Bayesian information criterion values should be lower to indicate a more appropriate model. Bayesian information criterion (BIC) was calculated as follows [37]:
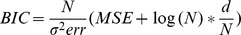
where 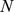
is the overall number of data points, 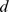
is the number of model parameters, 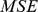 is the mean squared error and 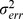
is the variance of the error.

**Table 2:** Best fit parameters of simulated slow-phase eye velocity and perceived rotational velocity curves.

	cerebellar patients	healthy controls	p-value
***unconstrained fit***			
*yaw rotations*			
*τ_C_*	4.2±1.1	5.0±1.2	p = 0.07
*τ_VSM (eye)_*	14.6±3.8	17.4±4.4	p = 0.10
*τ_VSM (perception)_*	14.4±3.7	13.9±7.1	p = 0.81
*pitch rotations*			
*τ_C_*	4.0±1.0	4.8±1.0	p = 0.09
*τ_VSM (eye)_*	8.8±4.3	5.3±3.2	p = 0.06
*τ_VSM (perception)_*	9.7±4.6	7.4±5.3	p = 0.29
***constrained fit***			
*yaw rotations*			
*τ_C_*	4.4±1.3	5.1±1.3	p = 0.18
*τ_VSM_*	14.4±3.8	15.3±4.2	p = 0.59
*pitch rotations*			
*τ_C_*	4.4±1.4	5.0±1.3	p = 0.30
*τ_VSM_*	9.3±4.5	6.0±3.0	p = 0.06

Best fit parameters of simulated slow-phase eye velocity and perceived rotational velocity curves in cerebellar patients and in age-matched healthy obtained with and without constraining the velocity-storage time constant (***τ_VSM_***). Values are means ±1SD. τ*_C_*: estimated peripheral semicircular canal time constants; τ*_VSM_*: estimated central velocity storage time constants.

**Figure 2:**
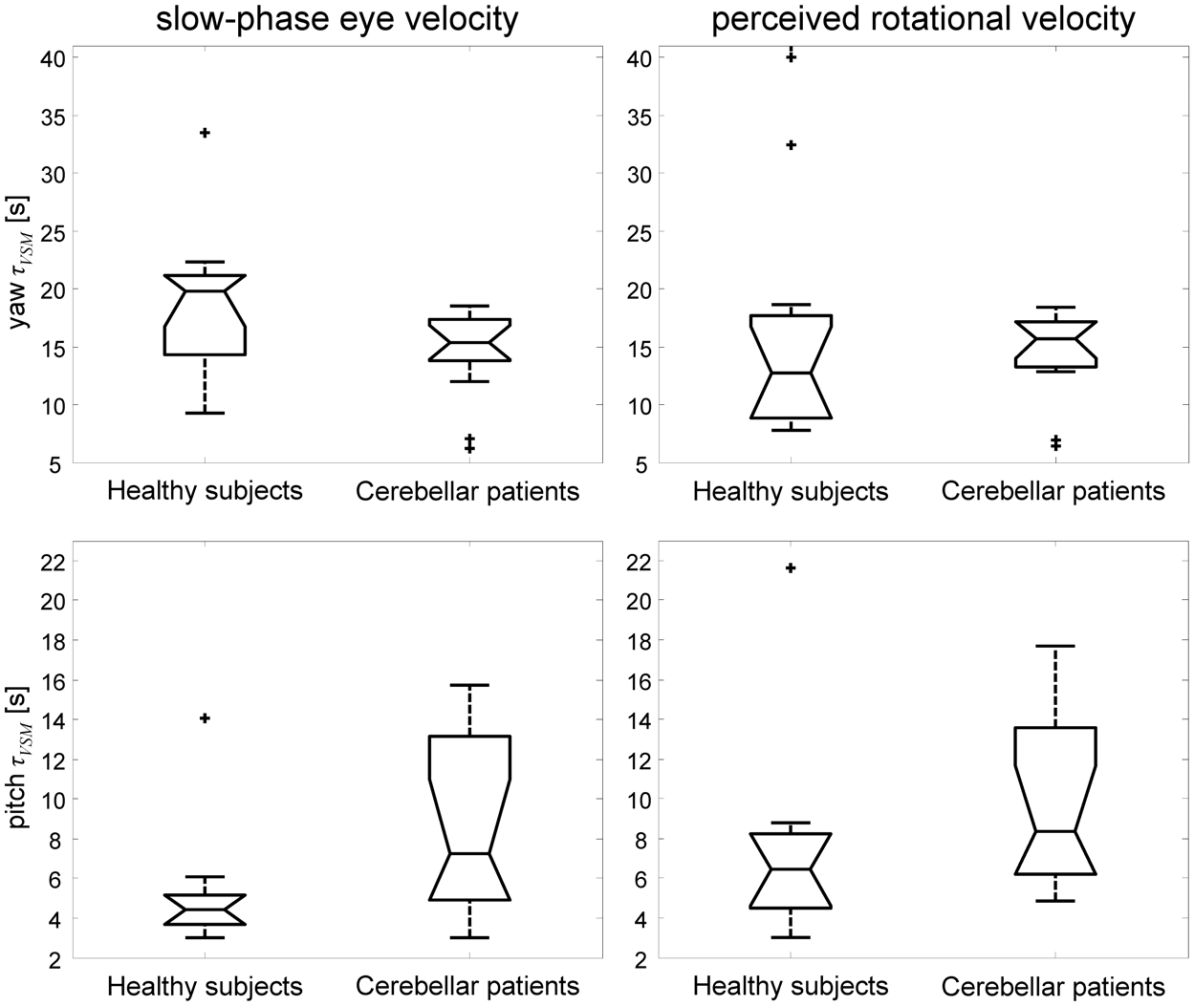
Comparison of velocity storage time constants of reflexive eye and perceptual responses in healthy subjects and cerebellar patients. Box plot representation of velocity storage time constant (*τ_VSM_)* estimates in healthy subjects and cerebellar patients. Unconstrained model fit, i.e. fitting procedure with *τ_VSM_* free to change for reflexive eye and perceptual responses. Note the different time scales for yaw and pitch rotations. Although no significant difference was found between *τ_VSM_* for reflexive and perceptual responses, values estimated from pitch responses (left and right bottom graphs) show a larger *τ_VSM_* spread in patients compared to healthy subjects.

**Figure 3:**
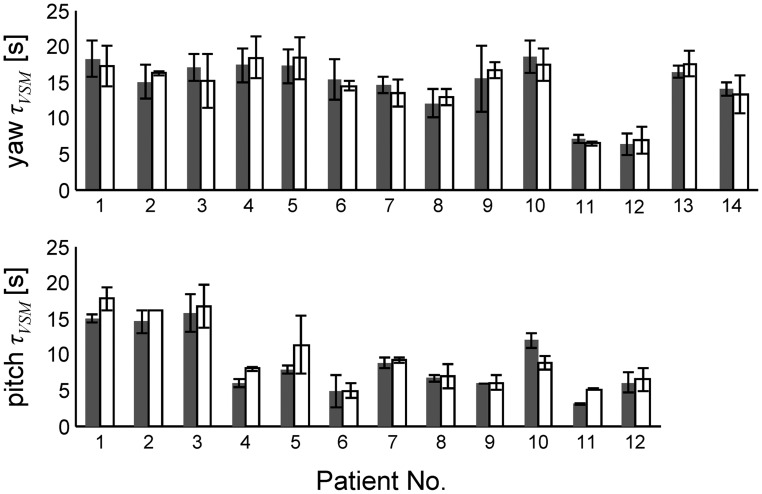
Velocity storage time constants of reflexive eye and perceptual responses. Comparison of the time constants (mean ± SD) describing the velocity storage activity (*τ_VSM_*) between slow-phase eye velocity (gray bars) and perceived rotational velocity (white bars) obtained by the model when letting *τ_VSM_* free to change. Each block of two bars represents the results in one subject. Two patients were rotated about the earth-vertical yaw axis only, because they reported motion sickness during earth-vertical pitch rotations.

## Results

As outlined previously (see Figure 1), slow-phase eye velocity and perceived rotational velocity responses to velocity steps of earth-vertical yaw and earth-vertical pitch rotations were fitted by the sum of two exponentials: one with a time constant accounting for the peripheral, i.e. semicircular canal (*τ_C_)*, and another with a time constant accounting for the central, i.e. velocity storage (*τ_VSM_)*, dynamics.

Slow-phase eye velocity and perceived rotational velocity responses were first fitted by letting *τ_VSM_* free to change (see *Data analysis* in Methods for further details). This allowed us to explore the relation of *τ_VSM_* of eye movements and *τ_VSM_* of perception responses without assuming any shared central processing. Recall, that, however, as a logical consequence of the simultaneous recording of eye movement and perception data, we constrained the model to use the same semicircular canal time constant *τ_C_* for fitting slow-phase eye velocity and perceived rotational velocity. Estimated model parameters are presented in Table 2 (see unconstrained fit). For comparison, values from age-matched healthy controls are also provided. Time constants of all samples were normally distributed and no statistical differences between patients and healthy controls were found during both yaw and pitch rotations (paired t-test p>0.1).

**Figure 4:**
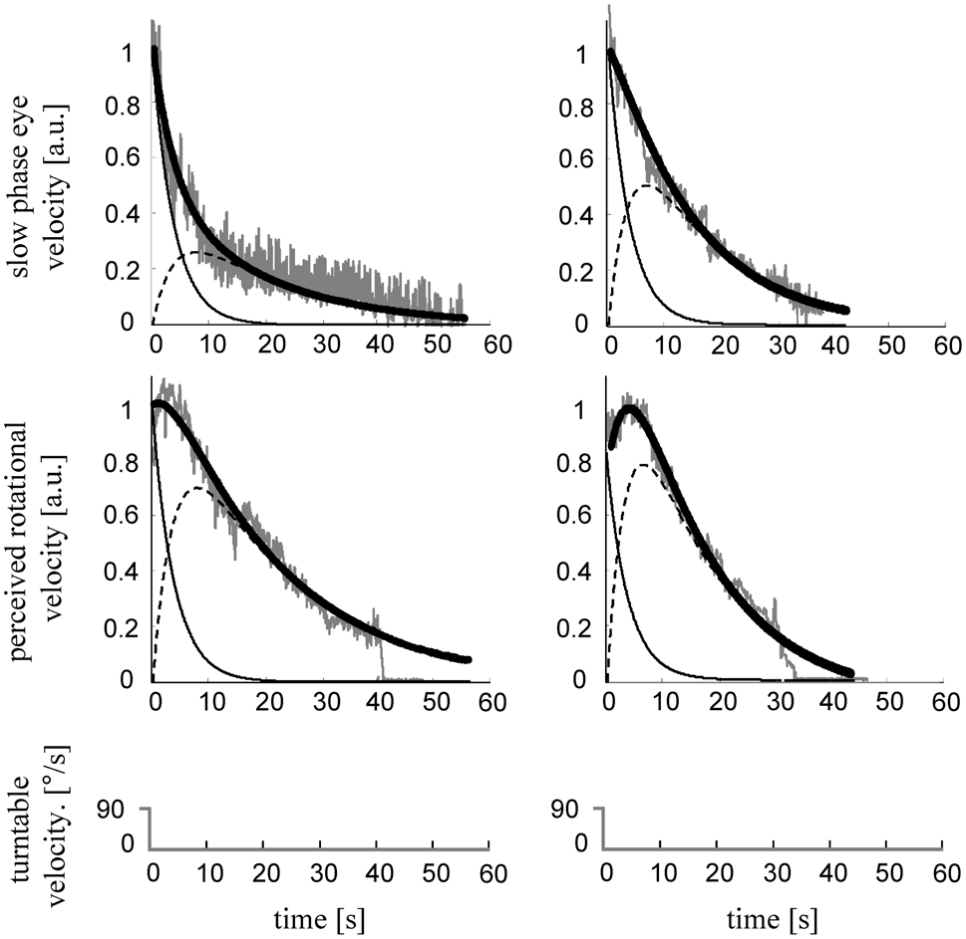
Variability of reflexive eye and perceptual responses in two cerebellar patients during yaw rotations. Slow-phase eye velocity and perceived rotational velocity responses with corresponding simulated curves in one patients (patient no. 8 [left column]) and one healthy subject (right column) after the sudden stop from an earth-vertical yaw rotation.

**Figure 5:**
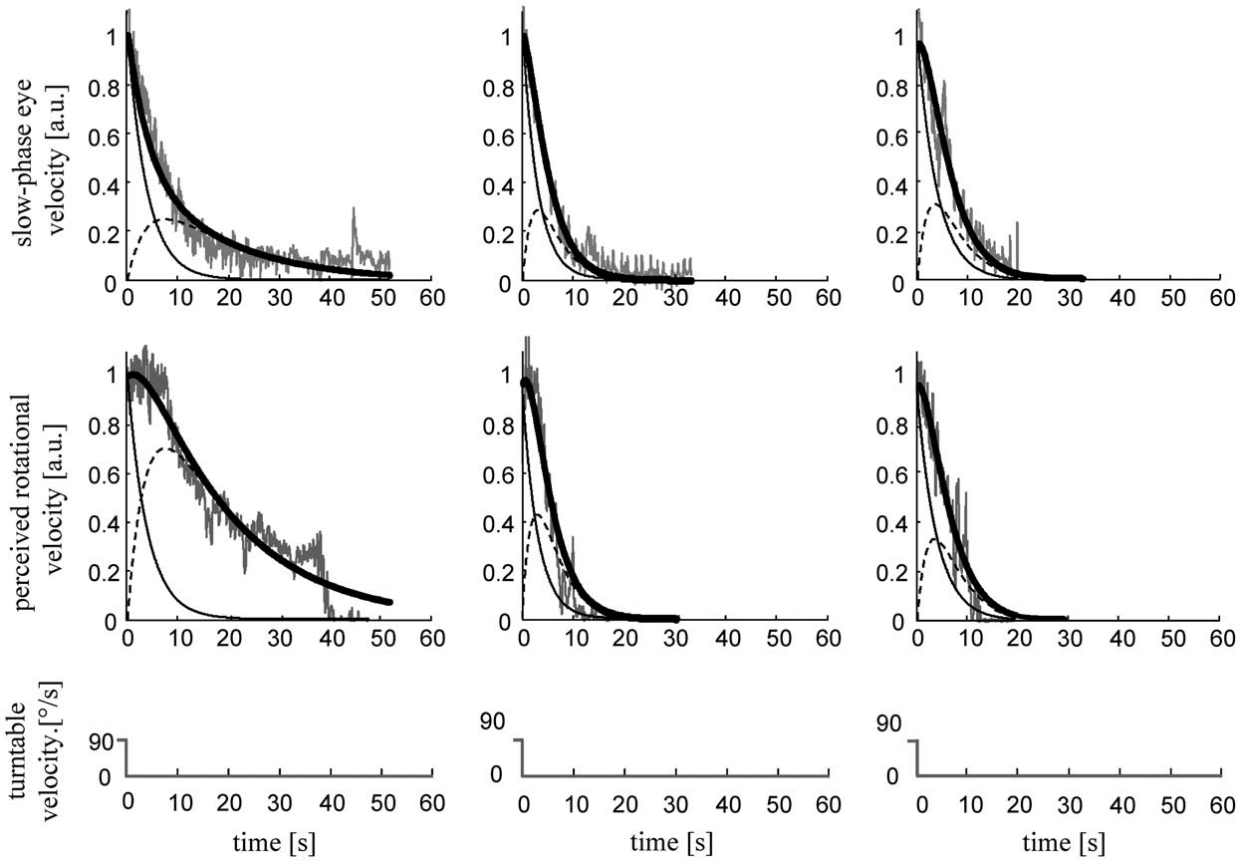
Variability of reflexive eye and perceptual responses in two cerebellar patients during pitch rotations. Slow-phase eye velocity and perceived rotational velocity responses with corresponding simulated curves in two patients (patient no. 2 [left column], patient no. 7 [middle column]) and one healthy subject (right column) after the sudden stop from an earth-vertical pitch rotation. The two patients demonstrate the wide spread of reflexive and perceptual responses observed during earth-vertical pitch rotations.

**Table 3:** Ratios of best fit gain parameters of simulated slow-phase eye velocity and perceived rotational velocity curves.

	cerebellar patients	healthy controls	p-value
*yaw rotations*			
***R*** *_vor_*	0.20±0.11	0.15±0.07	p = 0.17
***R*** *_perc_*	0.48±0.30	0.30±0.14	p = 0.07
*pitch rotations*			
***R*** *_vor_*	0.22±0.16	0.10±0.07	*p = 0.03
***R*** *_perc_*	0.45±0.26	0.35±0.18	p = 0.30

Ratios of best fit gain parameters obtained with constrained velocity-storage time constant (***τ_VSM_***). Values are means ± 1SD. *R_vor_* and *R_perc_*: ratio of indirect pathway gain to direct pathway gain estimated for slow-phase eye velocity and perceived rotational velocity, respectively.

Figure 2 provides a graphical representation of *τ_VSM_* values between patients and healthy subjects. Note that even though overall *τ_VSM_* was not statistically different between both groups, during pitch rotations *τ_VSM_* are spread over a wider range in the cerebellar patients.

The model function used to fit the data has previously been shown to be accurately applicable in healthy subjects [13]; its performance in the pathophysiological condition of cerebellar atrophy, however, has still to be proven. The goodness of fit (variance-accounted-for) in cerebellar patients for slow-phase eye velocity and for perceived rotational velocity was 0.83±0.09 and 0.87±0.07 during yaw rotations, and 0.80±0.16 and 0.89±0.06 during pitch rotations, respectively. Thus, also in cerebellar patients, self-motion perception and reflexive eye movement curves were fitted well by the model.

*τ_VSM_* of slow-phase eye velocity and perceived rotational velocity during trials of earth-vertical yaw and during trials of earth-vertical pitch rotations covaried in all cerebellar patients (Pearson correlation coefficient between rVOR *τ_VSM_* and perception *τ_VSM_*: 0.95 for yaw and 0.93 for pitch, p<0.001), indicating a possible link between the central time constant *τ_VSM_* of slow-phase eye velocity and perceived rotational velocity. Figure 3 shows *τ_VSM_* of slow-phase eye velocity and perceived rotational velocity (mean ± SD of all yaw and pitch traces) in individual patients.

We subsequently constrained the model to use the same *τ_VSM_* for fitting slow-phase eye velocity and perceived rotational velocity traces. Estimated model parameters with constrained *τ_VSM_* are presented in Table 2 (see constrained fit). For comparison, values from age-matched healthy controls are also provided. As for unconstrained *τ_VSM_* parameters, time constants of all samples were normally distributed and no statistical differences between patients and healthy controls were found during both yaw and pitch rotations (paired t-test p>0.1).

Similar to the unconstraint model condition, we found that cerebellar eye velocity and perceptual curves were fitted well by the model (variance-accounted-for during yaw rotations: 0.82±0.10 for slow-phase eye velocity and 0.86±0.05 for perceived rotational velocity; during pitch rotations: 0.80±0.13 for slow-phase eye velocity and 0.88±0.07 for perceived rotational velocity). The eligibility of the model when reducing the number of parameters by using the same *τ_VSM_* was additionally evaluated using the Bayesian information criterion (see *Statistical analysis* in Methods). With the model constrained to use the same *τ_VSM_* for fitting slow-phase eye velocity and perceived rotational velocity traces, Bayesian information criterion over all patients was 89±17 for yaw and 58±15 for pitch rotations, respectively. Such Bayesian information criterion values were not significantly different from those obtained when allowing the optimization procedure to vary *τ_VSM_* values for fitting slow-phase eye velocity and perceived rotational velocity (Bayesian information criterion values: 103±27 for yaw and 66±16 for pitch rotations; paired t-test for values obtained during yaw and pitch rotations: p>0.1).

Figure 4 and 5 show slow-phase eye velocity and perceived rotational velocity traces during yaw and pitch rotations in typical cerebellar patients and in a healthy age-matched subject. Figure 5 moreover highlights the wide spread of reflexive and perceptual responses found for pitch rotations in cerebellar patients.

From both figures one can also infer that the main characteristics of slow-phase eye velocity and perceived rotational velocity responses were similar in our patients and in healthy subjects. Specifically, slow-phase eye velocity showed an almost immediate rise followed by a slower decay approximating a single exponential curve, while perceived rotational velocity rise is followed by a plateau-like phase. The latter is especially evident in patients having a long *τ_VSM_,* such as the one shown in the first column of Figure 5. We previously found and explained this plateau-like behavior by proposing an increased relative weighting of the central velocity storage activity (for details about the qualitative description of reflexive and perceptual responses see [13]).

Values of gain ratios of the velocity storage component to the semicircular canals component in cerebellar and healthy subjects are provided in Table 3. Note that only arbitrarily scaled gains can be defined for perception of rotational velocity using a magnitude estimation method as done in this study [38]. Therefore the fitting procedure allows only to draw conclusions about the relative gain ratio between the direct, i.e. semicircular canals, and the indirect, i.e. velocity storage, pathways. A significant increase of the relative strength of the perceptual velocity storage pathway can be, nevertheless, seen in cerebellar patients for both yaw and pitch rotations and is in line with healthy subjects (see [13]).

## Discussion

In patients with chronic degeneration of the vestibulo-cerebellum due to hereditary or sporadic disease, we studied the vestibulo-ocular reflex and self-motion perception after a sudden stop from a sustained rotation about the earth-vertical axis (angular velocity step response) while seated either in the upright position (earth-vertical yaw rotation) or lying on the left side (earth-vertical pitch rotation). We aimed to investigate whether the loss of the regulatory control of the brainstem velocity-storage mechanism by midline cerebellar structures due to cerebellar atrophy would induce similar changes in rotational self-motion perception and in reflexive eye movements. Such finding would emphasize a possible sharing of the velocity storage mechanism between the rotational vestibulo-ocular reflex (rVOR) and self-motion perception. Results from this study support that hypothesis.

We found that reflexive eye movement and self-motion perception responses to an angular velocity step behaved similarly. By using a model originally developed for reflexive eye movements [10], [33] and recently adapted for explaining perceptual responses in healthy subjects [13], we specifically were able to accurately fit both rVOR and self-motion perception and observed a co-variation of the estimated reflexive and perceptual central time constants, these latter representing the velocity storage activity (see Fig. 1). When restraining the model to use the same central time constant for reflexive and perceptual responses, moreover, fitting accuracy was maintained, which reinforces our hypothesis about a possible common velocity storage mechanism in reflexive eye movements and self-motion perception.

A correlation of vestibulo-ocular and vestibulo-perceptual responses has previously been shown by Bronstein et al. in a similar population of patients with midline cerebellar degeneration [39]. While Bronstein et al. [39], however, determined the overall decay time constant of reflexive and perceptual responses, the present study provides a deeper insight into the possible role of the brainstem velocity storage mechanism itself. Specifically, the model-based approach allowed us to separate the peripheral (i.e. semicircular canal) and central (i.e. velocity storage) dynamics that contribute to the rVOR, and, thus, to estimate the parameters determining the contribution of velocity storage mechanism. The fact that perceptual responses could be accurately simulated by the same model architecture that was originally developed for the rVOR and – even more importantly – by restraining the model to use the same central time constant as for reflexive eye movement responses enforces our hypothesis of a common brainstem velocity storage mechanism in perceptual and vestibulo-ocular responses. The presence of similar rotational response dynamics reflecting velocity storage properties in thalamic and vestibular nuclei neurons [40], [41], [42], [43], [44], moreover, underpin our assumption that – at least for angular velocity step stimuli – a further perceptual, presumably cortical, processing is not necessary. Of course, we cannot rule out the possibility that different neuronal subgroups within the velocity storage mechanism contribute to reflexive and perceptual responses, as the velocity storage constitutes a network of neurons within the brainstem and cerebellum. The fact that a plateau-like behavior, quantitatively reflected by the significant difference in gain ratios between eye and perceptual velocities, was found only in perceptual but not in eye movement responses at least allows such speculation. At this point of time, in fact, we simply can provide evidence that the same model architecture holds for reflexive eye movements and perceptual responses. Further studies investigating perceptual responses in animals with single cell recordings will possibly shed further light on this conundrum (see [45] for a recent review).

We did not find any significant difference in the duration of reflexive eye movements and sensation in our patients compared to healthy age-matched subjects. This contrasts Bronstein et al. findings of a shortening of the vestibulo-ocular and vestibulo-perceptual responses compared to healthy controls. It is conceivable that this discrepancy might originate from subtle differences in the populations of patients, e.g. in etiology, disease duration, and region predominantly affected by cerebellar atrophy. Regarding this latter, in fact, ablation in rhesus monkeys point to a separate control of the horizontal and vertical/torsional rVOR time constants by different regions (specifically the lateral and central portions) of the nodulus and ventral uvula [23]. Moreover, as already outlined in the Introduction, studies in primates and in humans support the assumption that lesions along different neural structures of the vestibulo-cerebellum may cause somewhat opposite effects on the rVOR duration [15], [17], [23], [26], [27], [28], [29].

A last remark concerns the finding of a wide spread in the duration of reflexive and perceptual responses observed during earth-vertical pitch rotations in our patients (see Figure 2 and 3). Specifically, in three patients, reflexive and perceptual central velocity storage time constants were even twice the mean central time constants found in our healthy control group. Presumably, such prolonged time constant resulted from a loss of the suppressive effect of the vestibulo-cerebellum on the velocity storage network, known to occur in physiological conditions [15], [46], [47], [48]. Interestingly, moreover, motion sickness was foreground in those three patients during earth-vertical pitch rotations. Precisely how motion perception is linked to the velocity storage network is still unknown. rVOR habituation, a phenomenon producing the shortening of the time constant of the rVOR and depending on the integrity of the vestibulo-cerebellum [46], however, has been shown to decrease the susceptibility to motion sickness in humans as recently suggested in a study on healthy subjects [49]. Thus, although the exact nature and interplay between perception, the velocity storage mechanism and the vestibulo-cerebellum remains to be elucidated, our study evidences a crucial contribution of the velocity storage mechanism to perceptual control [19]. It furthermore underlines the important role of the vestibulo-cerebellum in the processing of motion perception [49], [50], [51] and provides support to the discussion about the role of the cerebellum in non-motor higher cognitive, behavioral and affective functions coined by Schmahmann and Sherman (1998) as the ‘cerebellar cognitive affective syndrome’ [52].
